# Human primary mixed brain cultures: preparation, differentiation, characterization and application to neuroscience research

**DOI:** 10.1186/s13041-014-0063-0

**Published:** 2014-09-16

**Authors:** Balmiki Ray, Nipun Chopra, Justin M Long, Debomoy K Lahiri

**Affiliations:** Laboratory of Molecular Neurogenetics, Institute of Psychiatric Research, Department of Psychiatry, Neuroscience Research Center, Indiana University School of Medicine, 320 W. 15th Street, Indianapolis, Indiana 46202 USA; Department of Medical and Molecular Genetics, Indiana University School of Medicine, Indianapolis, Indiana 46202 USA

**Keywords:** Alzheimer, Amyloid, Cholinergic, Dopaminergic, Development, Glutamatergic, Human brain culture, CNS, Confocal microscopy

## Abstract

**Background:**

Culturing primary cortical neurons is an essential neuroscience technique. However, most cultures are derived from rodent brains and standard protocols for human brain cultures are sparse. Herein, we describe preparation, maintenance and major characteristics of a primary human mixed brain culture, including neurons, obtained from legally aborted fetal brain tissue. This approach employs standard materials and techniques used in the preparation of rodent neuron cultures, with critical modifications.

**Results:**

This culture has distinct differences from rodent cultures. Specifically, a significant numbers of cells in the human culture are derived from progenitor cells, and the yield and survival of the cells grossly depend on the presence of bFGF. In the presence of bFGF, this culture can be maintained for an extended period. Abundant productions of amyloid-β, tau and proteins make this a powerful model for Alzheimer’s research. The culture also produces glia and different sub-types of neurons.

**Conclusion:**

We provide a well-characterized methodology for human mixed brain cultures useful to test therapeutic agents under various conditions, and to carry forward mechanistic and translational studies for several brain disorders.

**Electronic supplementary material:**

The online version of this article (doi:10.1186/s13041-014-0063-0) contains supplementary material, which is available to authorized users.

## Background

Primary brain and neuron cultures are used for neurobiological, neurodevelopmental, pharmacological and toxicological studies in various neuropsychiatric disorders. To date, the majority of the neuron cultures are prepared from embryonic rodent brains [1,2] and very few from adult rodent brain [3,4]. Rodents are preferred for preparing primary cultures because of availability, lower expense and less stringent regulations. However, rodents normally do not experience human-like neurodegenerative disorders such as Alzheimer’s disease (AD) or Parkinson’s disease (PD). Additionally, molecules involved in important pathological events (such as amyloid-β or Aβ and α-synuclein) in human neurodegenerative disorders are structurally different in rodents. Many continuous cell lines, such as human neuroblastoma or rat pheochromocytoma (PC12), are used as surrogate models for mature neurons by stimulating neuronal-type differentiation of the culture. However, these cancer cells may behave differently than CNS neurons. Recently, major emphasis has been given to differentiate human neurons from induced pluripotent stem cells (iPSC). However, this method could be expensive, time consuming and overall, the iPSCs are artificially transduced with several transcription factors. In the past, efforts have been made to prepare neuron cultures from the fetus of nonhuman primates [5,6], but the stringent regulation and scarcity of nonhuman primate colonies are major obstacles to performing such cultures on a regular basis. We have recently developed and characterized a primary neuron culture derived from the parenchyma of legally aborted human fetal brains. A small number of research groups have previously reported the use of primary human fetal neuron cultures in their studies [7–15]. While fetal cultures have been used to examine the effects of estrogen [12], and laminin [16], a detailed protocol for preparing, maintaining and characterizing such cultures is lacking. Neuroprogenitor cells from adult donors’ brains are also available from commercial sources, or have been cultured [15,17,18], but are difficult to grow and also lack detailed characterization. It is possible to generate astrocytic and oligodendrocytic [15] cultures from fetal tissue as well. However, there remains a dearth of publications that outline the culture of a mixed neuronal culture derived from human tissue. One such article [13] was able to demonstrate neuronal and glial positive staining and the formation of synaptic boutons. Notably, we are able to show quantitation of the immunostaining and measure physiological activity of our neurons using flow cytometry and calcium imaging respectively. Moreover, the applicability of our culture to studying neurodegenerative disorders has been shown by our ability to demonstrate the presence of several neuronal and synaptic markers and of significant levels of Alzheimer’s Aβ peptides, and the transfectability of small RNA species, such as siRNAs. Therefore, we are able to outline a protocol for a culture that displays: a) both neuronal and glial characteristics; b) physiological activity; c) the potential for extensive utility across neuroscience. We have successfully employed the primary culture system described in this protocol in our research workflow [19].

## Results and discussion

### Major differences between primary human and rodent embryonic neuron culture

The human brain is approximately sixty times larger than the mouse brain. The majority of the neurons obtained from fetuses (90–110 days gestational age) are immature. Development of the human cerebral cortex begins at the end of the first trimester and continues through the second trimester [20]. Since this protocol utilizes brain tissue from fetuses at a gestational age slightly beyond 90 days (i.e. first trimester), the majority of the neurons are immature, and a good proportion of isolated cells are neuroblast and/or neural progenitor (NPCs) in nature. At the initial phase of the cultures, the cells co-express markers of both neuronal and glial origin, consistent with the immature nature of cells in culture. After a longer period of culture, both mature neurons and glia are present. This is an important feature of this culture and contrasts with primary embryonic rodent cultures, where the culture is generally prepared from embryonic day 16–18 (E16-E18) brain; equivalent to the third trimester of human pregnancy. Therefore, these rodent brains contain many more post-mitotic neurons than the human fetal neuron cultures. We have characterized the human culture and observed mature neurons and glia in the culture from *day in vitro* (DIV) 20 onwards. Around this point of time, neurons and glia can be visualized separately by immunofluorescence staining using specific neuronal and glial markers.

### Generation of viable cells that stain for neuronal and glial markers

**Figure 1:**
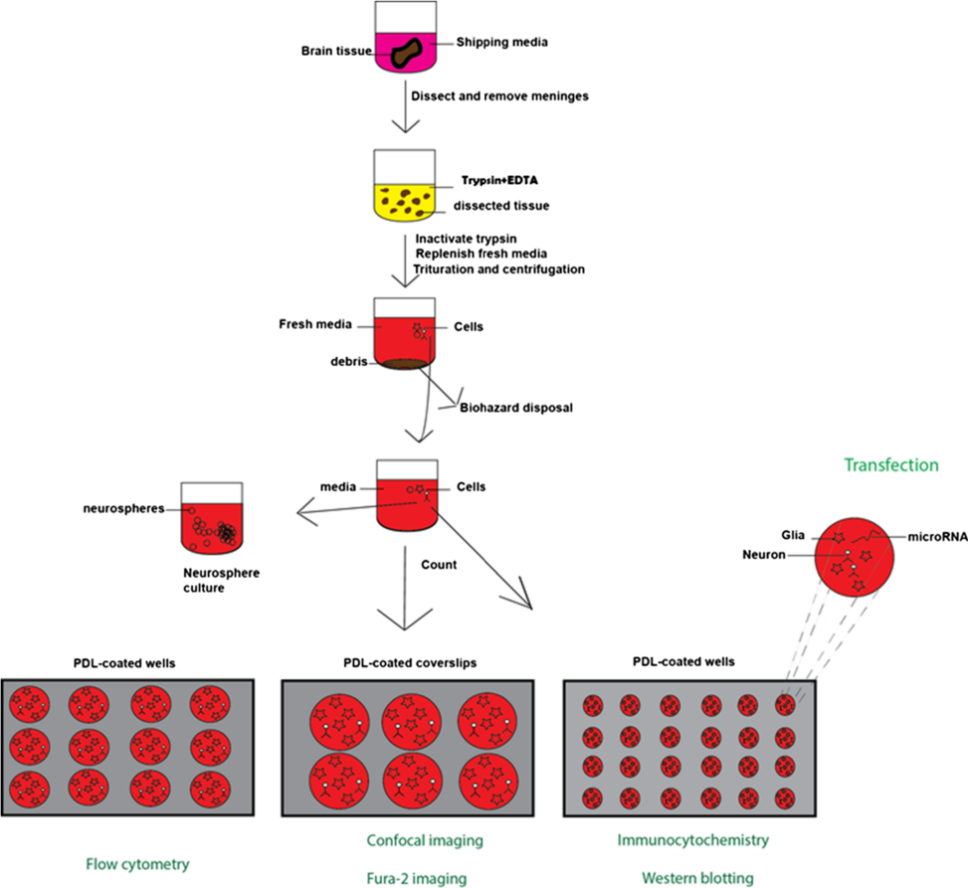
**Schematic diagram summarizing the major steps involved in the procedure to prepare human neuron culture.** Also shown are 6-well, 12-well and 24-well formats that can be used for different biochemical and physiological studies pertaining to neuroscience. The inset shows a schematic of miRNA transfection into the culture.

**Figure 2:**
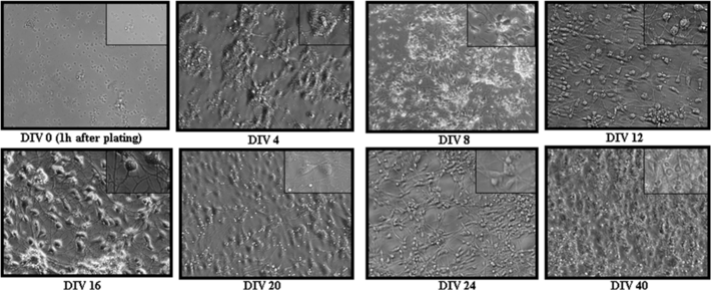
**Phase contrast pictures (20 X magnification) showing the gross morphology of the cells at different time points of the culture.** Rounded cell bodies can be seen soon after plating the cells. Discrete cell clumps are visible at the early time points of the culture. Significant proportions of cells at this time point represent NPCs, which later on give rise to neurons and astrocytes. Abundant numbers of cells are present even at DIV40 of the culture. The insets are representative portions from the same image that have been enlarged.

**Figure 3:**
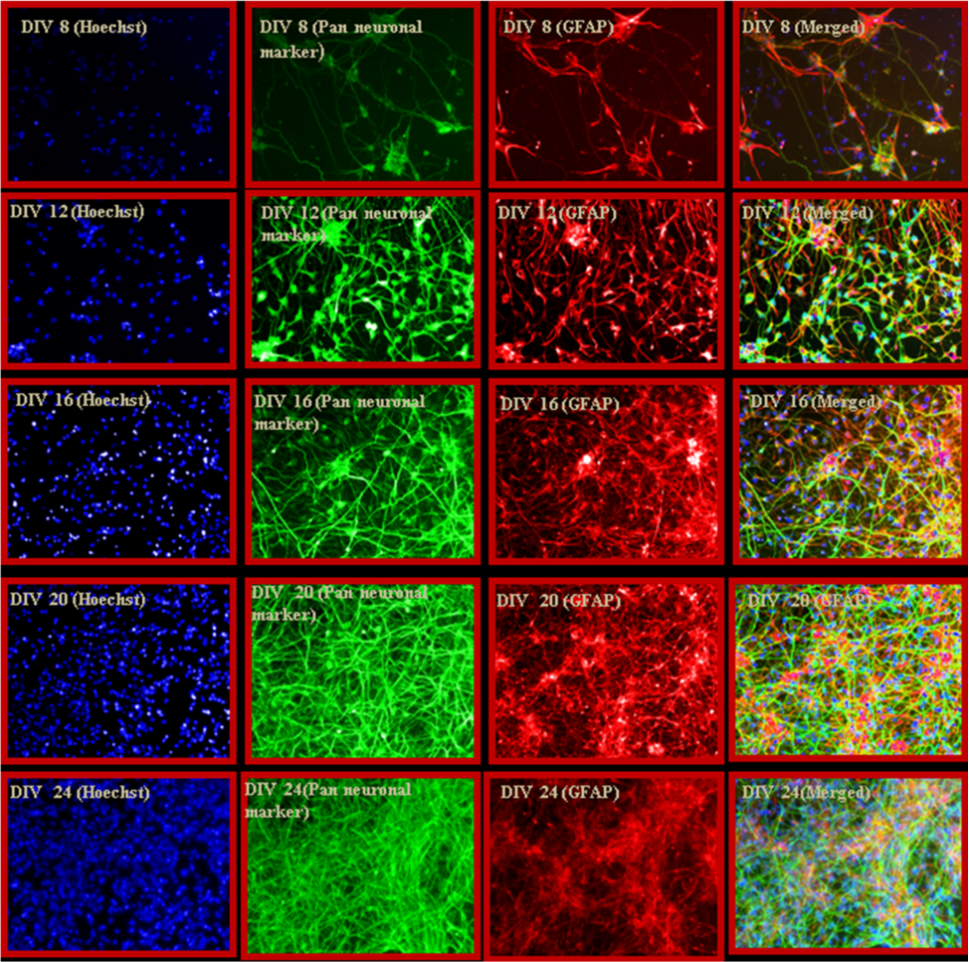
**ICC study of the culture using neuronal and glial-specific marker shows the presence of both the phenotypes in the culture.** Interestingly, cells at the initial period of the culture are labeled with both neuronal and glial markers indicating newly generated or immature cells. However, the cells become mature at the later time points (DIV 20–24), when neurons and glia are stained separately by pan-neuronal and GFAP antibodies, respectively.

We outline a procedure (Figure 1) to show the plating of primary human brain culture. We confirmed that these cells were viable, in vitro, until at least DIV 40 (Figure 2) and that they continue to show cells that have neuronal morphology with a network of processes. In order to confirm the identity of these cells, we first performed immunocytochemistry using antibodies to neuronal and glial markers (Figure 3). We found that the culture contains both the aforementioned cell types. This is important, as it may provide an appropriate in vitro model for studying neurodegeneration and neurodevelopment.

### Effects of growth factors on neuronal viability and differentiation

**Figure 4:**
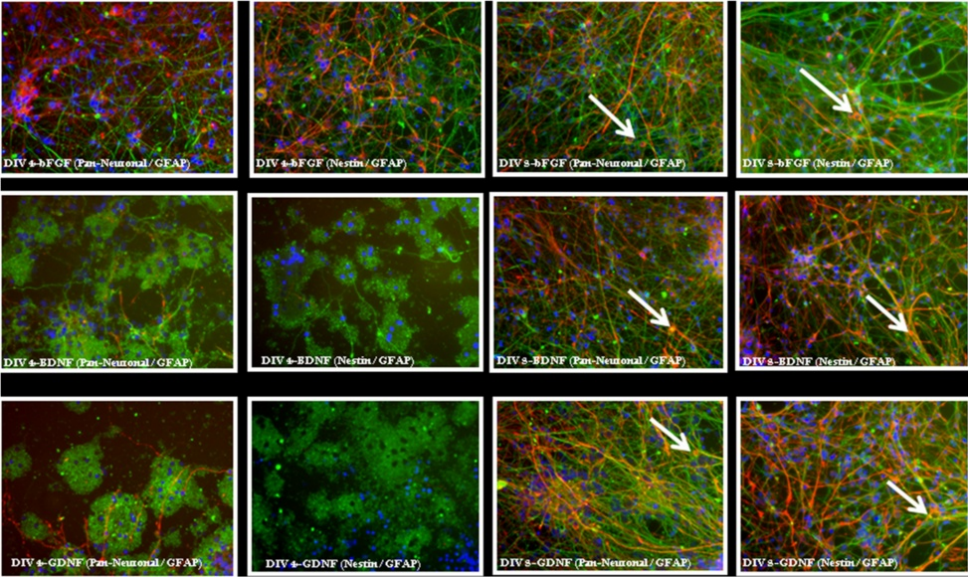
**Effects of growth factors in neuronal viability and differentiation.** To determine the requirement and effectiveness of different growth factors in neuronal survival and differentiation, we plated neurons with bFGF at DIV0 and changed complete media at DIV4. In the first experiment, at DIV4, different growth factors (bFGF, BDNF and GDNF) were added separately to the culture. A half-media change with the addition of respective growth factors was performed at DIV8, and the cells were fixed at DIV12. In the second experiment, cells received bFGF from DIV 0–8, thereafter media was aspirated off and fresh media containing bFGF, BDNF and GDNF were added separately. Cells were fixed at DIV 12. Fixed cells from both the experiments were permeabilized and then incubated with Pan-neuronal and GFAP or Pan-Neuronal and Nestin primary antibodies. Cells were incubated with appropriate fluorophore-conjugated secondary antibodies, washed off excess unbound antibodies, and immunofluorescent images were captured as stated in the text. Nuclei were stained by Hoechst dye. The figures were pseudocolorized using Adobe Photoshop CS4, where Pan-Neuronal-positive cells were colorized green, GFAP and Nestin-positive cells red and Hoechst were shown in blue. The figures depicted that cellular viability and differentiation were grossly decreased in the cultures that were supplemented by bFGF from DIV0-4, but received BDNF and GDNF from DIV4–12. However, cellular viability and differentiation were not affected in the group that received bFGF from DIV0–12. Contrarily, we have not noticed any alteration in cellular viability and differentiation in the cultures that were supplemented by bFGF from DIV0-8 and then supplemented by either bFGF, BDNF or GDNF from DIV8-12. These results indicate that supplementation by bFGF at the initial phase of the culture is essential for proper development and differentiation. Arrows indicate cell bodies that stain for both antibodies.

In order to prepare and maintain healthy neuronal cultures, we have tested several growth factors by observing their effects on neuronal differentiation. Basic fibroblast growth factor (bFGF) produces the best response in terms of long-term culture growth and differentiation. Previous studies have shown that FGF treatment promotes cortical neurogenesis by inducing proliferation of NPCs, resulting in an increased number and density of glutamatergic and GABAergic neurons [21]. Concomitantly, the presence of bFGF also increases the glial population by acting as a mitogen for glial precursor cells [22]. bFGF also induces neuronal differentiation and preserves the stem-cell population in the NPCs [23,24]. Indeed, nestin-positive cells (a well-known marker for neural stem cells [25]) are frequently observed in the culture described here (Figure 4, Additional file 1: Figure S1). We have observed diminished cellular viability and differentiation when BDNF and/or GDNF were added separately in the beginning (DIV 4) of the culture in the absence of bFGF. However, no significant alteration in culture character was noticed when BDNF (25 ng/mL) and/or GDNF (25 ng/mL) were separately added to the culture at a later time point (DIV 8), when the culture received bFGF from DIV 0–8 (Figure 4). We have also assessed the effects of other growth factors including nerve growth factor (NGF), glial cell-derived neurotrophic factor (GDNF) and other differentiation promoters, such as retinoic acid (RA) and forskolin, and noticed similar results (data not shown).

### Characterization of the mixed brain culture

**Figure 5:**
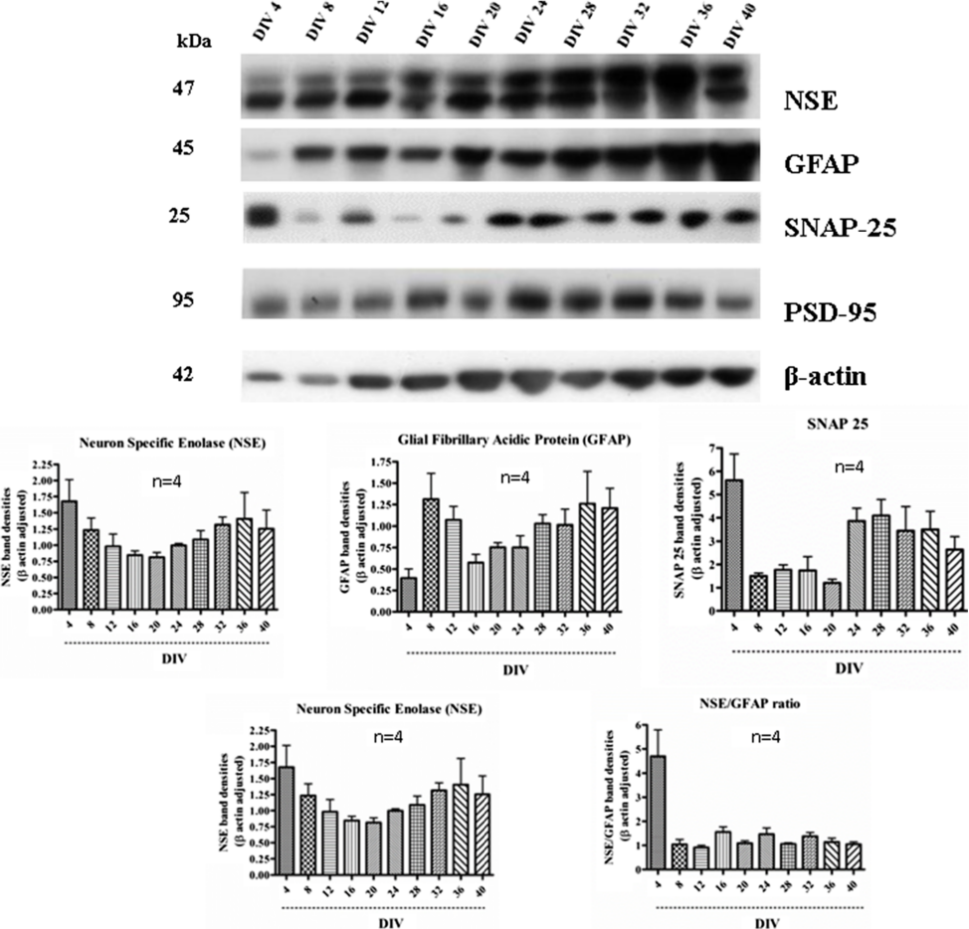
**Western immunoblotting of the cell lysates from cultures at different DIV shows presence of neuronal, glial and synaptic proteins (both pre-synaptic and post-synaptic proteins) at different time points of the culture.** Band densities of different proteins were scanned, quantified and normalized with β-actin bands. Levels of neuron specific enolase (NSE), a marker for neurons, and glial fibrillary acidic protein (GFAP), a glial marker, increase time-dependently in the culture. These may suggest a continual process of neuro- and gliogenesis in the culture. This finding is in accordance with the presence of NPCs (nestin positive cells) observed at all the time points of the culture. Notably, presence of synaptic markers (SNAP-25 and PSD-95) even at the later time points indicates non-degenerating nature of the culture.

**Figure 6:**
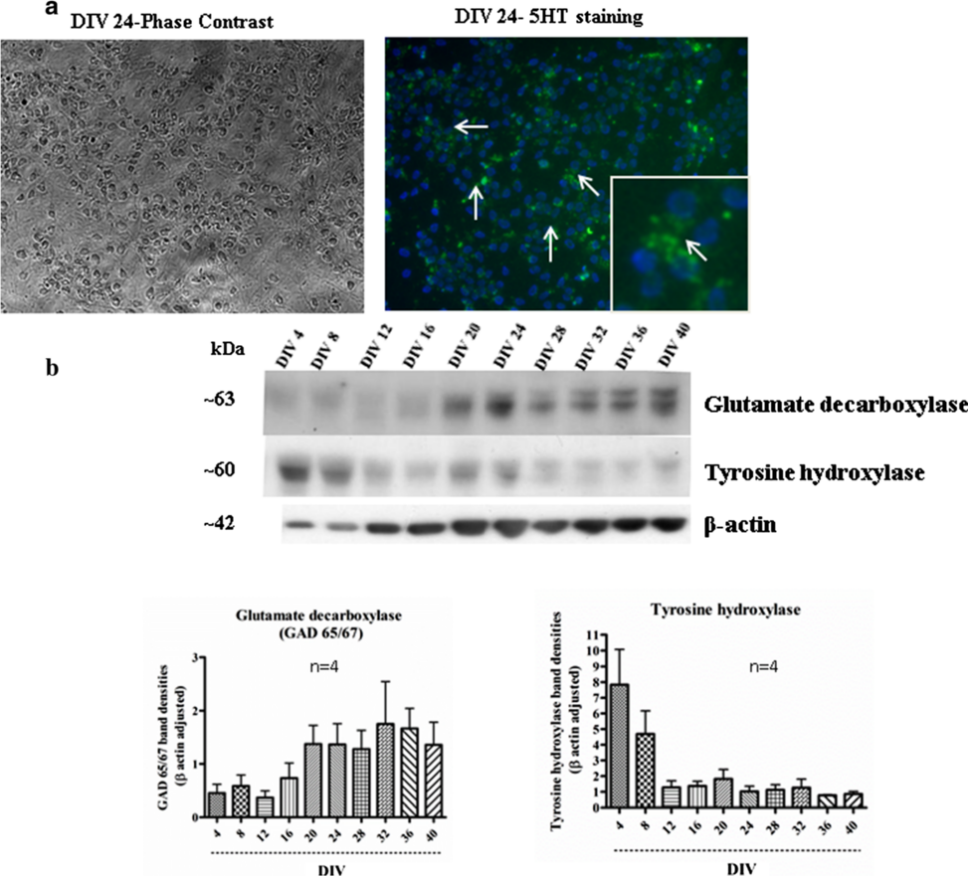
**Serotonergic and dopaminergic characterization of human mixed brain culture. a** Cells at DIV24 were fixed and ICC with 5-HT antibody revealed presence of serotonergic cells in the culture. The arrows indicate representative cells positive for 5-HT staining. The inset is an area magnified to better visualize 5-HT positive staining. **b** Western immunoblotting of the cell lysates indicates presence of GABAergic neurons (detected by glutamate decarboxylase antibody) in the culture. Number of GABAergic neurons gradually increase as the culture progresses. In contrast, dopaminergic neurons (detected by tyrosine hydroxylase antibody) are abundant in the initial time points of the culture and gradually decrease as the culture progresses.

**Figure 7:**
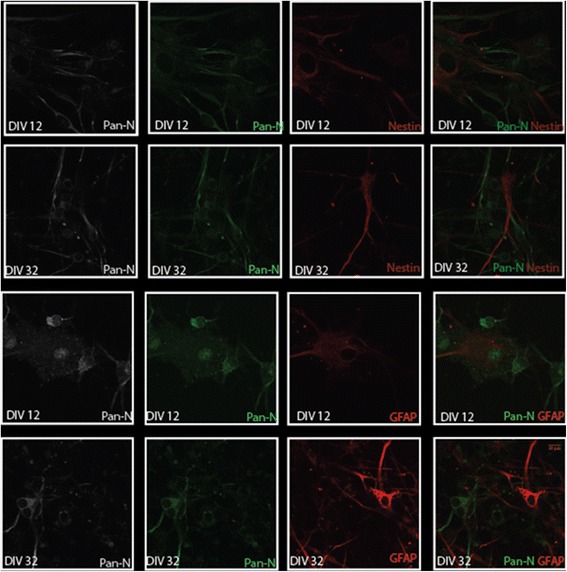
**Confocal imaging of Human mixed brain culture containing neurons, glia and neural progenitor cells.** At each time point, cells were fixed using 4% Paraformaldehyde and dual-labeled with FITC-conjugated Neuronal antibody cocktail (Pan-N, 1:1000) and a Cy3-conjugate of either a glial marker (GFAP, 1:5000) or a neuronal progenitor (Nestin, 1:500). Results at DIV 12, and DIV 32 are shown. Antibodies against Pan-N and or Pan-N and GFAP were used with appropriate secondary antibodies (donkey-anti mouse; 1:500 or Goat-anti rabbit, 1:2000). Confocal fluorescence microscopy was performed on an inverted IX81microscope fitted with a FV1000-MPE and laser launch with confocal excitation with three diode lasers (405, 559 and 635 nm) and an Argon laser (458, 488, 514 nm), controlled by Fluoview v3.0 (Olympus). All imaging was performed with a 60x 1.2 NA water immersion objective. The scale bar is in the bottom right panel, and is applicable to all images.

**Figure 8:**
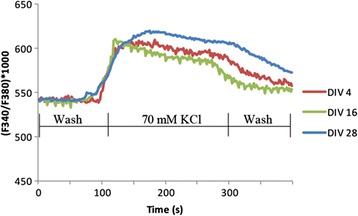
**Functional response of brain culture.** Cells were cultured on PDL-coated glass coverslips. Approximately 100 regions of interest (ROI) s were selected indiscriminately. The coverslips are loaded with Fura-2 dye and placed in standard extracellular solution (see methods). After depolarization with 70 mM KCl, another wash with standard extracellular solution is utilized to eliminate dead cells. The trace shown represents an average (3 experiments) of all cells that respond to KCl and the second wash. It is important to note that there were cells that did not respond to KCl which could be dead cells or non-neuronal cells. Fura emits at two wavelengths – 340 nm and 380 nm [26] and the ratio of these corresponds to the intracellular calcium. All three points show a calcium response after induced depolarization, suggesting that these are functionally active neurons.

**Figure 9:**
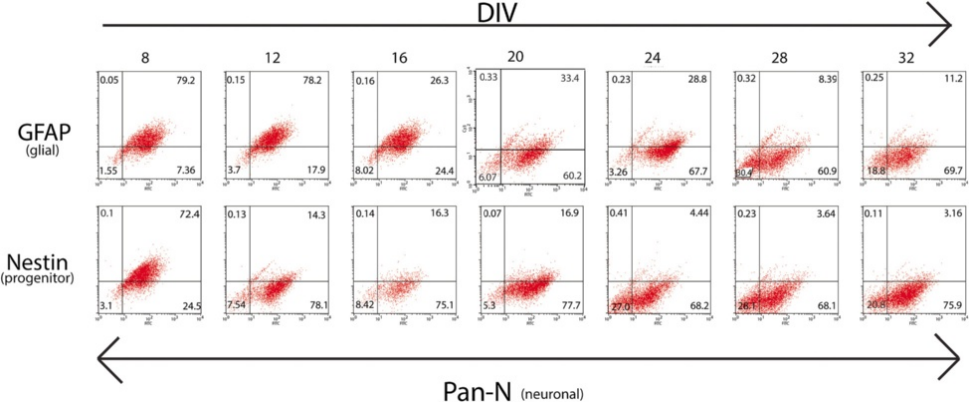
**An increase in neuronal population over time.** Human fetal brain (HFB) cells were grown as stated. At each time point, cells were fixed using 4% Paraformaldehyde and dual-labeled with FITC-conjugated Neuronal antibody cocktail (Pan-N, 1:1000) and a Cy3-conjugate of either a glial marker (GFAP, 1:5000) or a neuronal progenitor (Nestin, 1:500). Appropriate secondary antibodies were used. The values at the corners of the boxes refer to percent total of the cells gated for selection that were positive for the appropriate fluorescent-conjugated antibody. LL – Unlabeled cells. UL-Cells positive for Cy3-conjugated marker for either GFAP or Nestin. LR – Cells labeled with Pan-N. UR – Cells positive for either Pan-N and GFAP or Pan-N and Nestin. This data suggests that the stem-ness of the culture reduces over time and the neuronal phenotype beings to emerge.

In order to elucidate the molecular identity of our mixed culture, we used western blotting (Figures 5 and 6), confocal imaging (Figure 7), calcium imaging (Figure 8) and fluorescence activated cell sorter (FACS) (Figure 9). The human primary neuronal culture described here can serve as a relevant, rational and suitable tissue culture model for research on neurodegenerative disorders like AD, PD and other brain disorders. This culture contains measurable levels of synaptic (PSD-95, SNAP-25); glial (GFAP) and neuronal specific (NSE) proteins as evaluated by western blotting (Figure 5). Deposition of Aβ peptides and hyperphosphorylation of microtubule associated protein tau is the major hallmarks of AD. We have observed that this culture system produces significant amounts of Aβ (1–40) and Aβ (1–42), as assessed by a sensitive, human-specific ELISA. We have also measured AB at other time points [19]. At DIV 18, the amounts of Aβ (1–40) and Aβ (1–42) were measured to be 806.76 ± 212.02 pg/mL and 352.43 ± 95.95 pg/mL, respectively. We have also measured total and phospho-tau levels in the cell lysate samples by Western immunoblotting using specific antibodies. Total and phospho-tau levels were detected throughout the time points of the culture with highest peaks from DIV 28 onwards (Ray & Lahiri Unpublished data). The presence of tyrosine hydroxylase-positive neurons (Figure 6b) also makes this neuron culture appropriate for research related to PD. Serotonergic neurons and GABAergic neurons are also detectable, (Figure 6a and b) enabling this model to be employed in a wide variety of studies requiring these particular neuronal subtypes. Furthermore, the neurons present in the culture are suitable for electrophysiological study because of the expression of voltage-gated calcium channels (VGCC) as measured by Fura-2 AM experiments (Figure 8). The cells respond to depolarization by 70 mM KCl by allowing an influx of calcium, suggesting the presence of active VGCCs. We observed KCl-evoked Calcium influx at all three time points studied – DIV 4, DIV 16 and DIV 28 (Figure 8). The experiments show that these cells have a resting intracellular calcium level of 40 nM (data not shown). The slightly lower than expected intracellular calcium may be a consequence of the mixed cell culture that contains both mature and immature neurons.

Interestingly, the numbers of nestin and pan-neuronal co-labeled cells are higher in number at the initial phase of the culture (Figure 9) and gradually increase neuronal positive markers, suggesting an *in vitro* maturation process. Nevertheless, we observe presence of some nestin (Figures 7 and 9) positive cells during all time points of the maintained culture possibly explaining why we can maintain the culture for an extended period of time. Although we observe coexpression of neuronal and glial markers (Figure 9) during the early DIV, we see an increase in neuronal staining at the later stages of in vitro maturation. However, we do continue to see a small cluster of cells that continue to coexpress the two markers.

### Testing transfectability of nucleic acids in primary human brain culture

**Figure 10:**
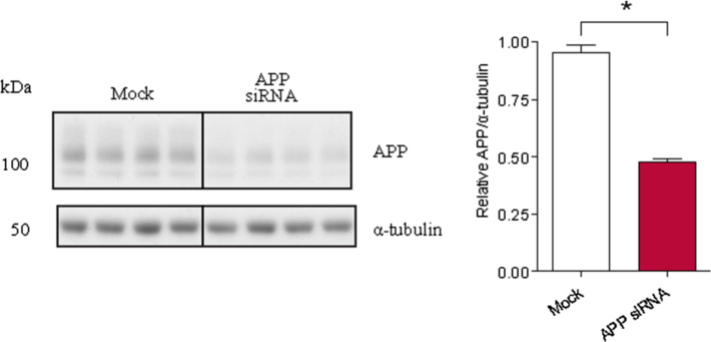
**Transfection of human fetal neuron culture using RNAiMAX transfection agent and APP siRNA demonstrates transfectability of cells.** Cells were transfected at DIV19 in a 24-well cell culture plate using 1.75 μL RNAiMAX (Invitrogen) and 20 nM APP siRNA (Applied Biosystems) as per manufacturer’s instructions. To improve clarity, we have drawn boxes to separate groups. Cell lysates were prepared 72 h post-transfection and APP levels (22C11, Millipore; Cat #MAB348) assayed by Western blot. APP levels were reduced by over 50% following transfection of APP siRNA. *p < 0.05 by Student’s t-test.

With appropriate maintenance, this neuronal culture can be kept viable for more than 40 DIV, thus making it a powerful tool for aging and/or toxicological research. In our hands, these cells are readily transfectable with small RNA constructs [27]. For example, we were able to knockdown ~50% of the amyloid precursor protein (APP) levels by transfecting this culture with APP siRNA at DIV 18 of the culture (Figure 10).

### Experimental design

This protocol describes an effective way to culture a viable mixed population of cells (both neurons and glia), which can survive *in vitro* for a prolonged period of time. Some important methodological alterations from primary rodent neuronal culture preparations were introduced to efficiently prepare such cultures for better neuronal yield and viability. An outline and salient features of the human primary neuronal culture preparation and progression is shown in Figure 1. Initially, the extracellular matrices and supporting stroma of human aborted fetal brains are significantly more abundant than in rodent brains, requiring an effective proteolytic digestion step. Meticulous dissection/processing of fetal brains to remove blood stained tissue is important to minimize the chance of contamination of the culture. It may be necessary to apply a broad-spectrum cocktail of antibiotics due to the inherent lack of sterility in the process of obtaining abortus material. The processed cell suspension (step 5 in Figure 1) can also be used for culturing neurospheres with appropriate media and growth factors [28]. Finally, this protocol can only be used after proper ‘Institutional Review Board’ (IRB) approval for the use of aborted human fetal tissue.

## Conclusion

We have successfully prepared human primary neuron cultures following our protocol and observed consistency in terms of cellular morphology, growth pattern, and neuronal maturation across cultures. The cells have demonstrated neurite sprouting from DIV 6–8. Cells at earlier time points aggregated in condensed clumps situated discretely across the plate. Major proportions of the cells were neuroprogenitors that gave rise to neurons and glia in due course of time. A subset of cells showed calcium influx in response to depolarization that are consistent with the presence of active VGCC. We observed that neurons in the culture matured no later than DIV 24. In the presence of bFGF, the culture survived for at least 40 days. To prepare such cultures, we used brains from aborted fetuses of gestational age between 85–110 days (Table 1). We also confirmed the transfectability of the cell culture using siRNA.

**Table 1:** **Total cell yield from brains of different gestational period**

**Culture replicate number**	**Gestational age (days)**	**Total cell yield (in million)**
1	108	141
2	105	77
3	108	115
4	98	124
5	96	121
6	104	130
7	94	113
8	101	124
9	101	145
10	103	118

### Materials and methods

#### Human fetal brain tissue

Human fetal brain tissue samples were obtained from aborted fetus of gestational age between 90–110 days. Aborted fetal brain tissues can be obtained from local abortion clinics or national distribution labs. In this study, we procured human fetal brain tissue samples from electively aborted legal aborted fetuses from the Laboratory of Developmental Biology, Seattle, WA following appropriate guidelines. The availability of the tissue is dependent on lab hours, donor visitation and the age of the samples desired. Coincidentally, all our HFN cultures were from male samples.

Handling and research utilizing human aborted fetal tissue requires prior approval from Indiana University Institutional Review Board (IRB) and should be in accordance with governmental regulation and policies. All individuals handling these tissues should be immunized against Hepatitis A and B and work in a Biosafety level 2 room. Additionally, these individuals should use great caution and all materials used should be autoclaved before disposal. Our protocol was approved by the IBC (Institutional Biosafety Committee) and IRB of Indiana University School of Medicine, Indianapolis, USA.

#### Preparation of shipping media

Hibernate E medium (Gibco, cat. no. A12476-01) is used to ship the brain tissue samples from the laboratory/clinic of origin. The Hibernate E medium is supplemented with regular 50X B27 (Gibco, cat. no.17504-044), 1 X antibiotics cocktail (Mediatech Inc., cat. no. 30-004-CI) and 50 mM Glutamax (Gibco, cat. no. 35050–061). Glutamax is light sensitive and should be stored appropriately at room temperature. The shipping media is also used to dissect and process brain tissue samples for the culture in ambient oxygen.

Brain tissues can be directly shipped from the abortion clinic (or appropriate centers) to the research laboratory in cold shipping media. The brain tissues are normally shipped in 15 mL PET tubes on ice containing at least 2.5 times more media volume than that of the brain tissue. The same medium is used for dissecting and processing the tissue before plating of the cells. It is important to prepare the culture within 24 h of brain tissue collection. Expedited, overnight shipment is recommended. The shipping media should be made fresh every 30 days. Any unused brain tissue should be bleached solution for 15 min. and then disposed in a biohazard disposal container.

#### Preparation of culture media

Neurobasal medium (Gibco, cat. no.12349-015) is used for the neuronal culture in the presence of 5% CO2. To culture neuronal cells, the Neurobasal medium is supplemented by 1X B27, 50 mM Glutamax, 1X antibiotics cocktail and recombinant human basic fibroblast growth factor 2 (bFGF) at a concentration of 5 ng/mL of the media (Gibco, cat. no. PHG0264). It is important reconstitute the growth factor (bFGF) properly before use. The reconstitution buffer generally contains 0.1% BSA as a carrier molecule. The reconstituted liquid should be passed through a 0.2 μm filter, aliquoted and stored at −20°C. Efforts should be made to avoid repeated freeze-thaw of the reconstituted bFGF. If the culture shows frequent signs of contamination, Normocin (InvivoGen, cat. no. ant-nr-1) can be added into the culture media at a concentration of 2 μL/ mL. The supplements mentioned above are added to the Neurobasal media and stored at 4°C for a month. bFGF is mixed to the required volume of media at the time of addition onto the wells. Some researchers add other growth factors, such as BDNF, GDNF, NGF or forskolin to induce neuronal differentiation [29]. However, we have observed optimum growth of the culture in the presence of bFGF alone.

#### Equipment and reagent setup

Water bath with orbital shaker: The water bath should be set at 37°C at least 1 h before the tissue dissection. The orbital shaker speed is to be set at 150 RPM. Plastic tube holders (designed to hold 50 mL tubes) should be placed at the bottom of the water bath to ensure that the tube holders uniformly shake at the same speed as the orbital shaker.

Silicon coating of glass Pasteur pipettes: Glass Pasteur pipettes’ (preferably 9 inch) should be fire-polished using a gas burner. One should make sure that the fire-polished tips are not too narrow (standard tip diameter should be between 0.75-1.25 mm). For silicon coating, Sigmacote (Sigma; cat. no.SL-2) is gently aspirated through the pipette using a dropper bulb aspirator. Once the Sigmacote reaches the top of the Pasteur pipette, the dropper bulb is removed from the pipette, allowing the liquid to drain down the pipette. In this way, 2–3 Pasteur pipettes are coated with Sigmacote. Coated Pasteur pipettes were dried for at least 30 min. under hood. Narrow portion of a separate Pasteur pipette was broken to make a wide-bore opening, fire-polished and siliconized in the same way as mentioned above. This pipette was used to transfer brain tissues.

Great caution is required in handling sharp materials. Sigmacote is highly flammable and should always be used under hood.

Centrifuge: The centrifuge was setup to the desired temperature (25-30°C) 2 h prior to the experiment. Rotors and tube holders were properly placed. Each tube was balanced and properly labeled.

#### Preparing tissue culture plates

The culture plates were coated with poly D-lysine/PDL (Sigma, cat. no. P7886). Reconstitution of powdered PDL was done in sterile water at 100 μg/mL, ran through sterile-filter, aliquoted in 50 mL polyethylene terephthalate (PET) tubes and stored at −20°C for at least 3 months. The required amounts of PDL were transferred onto every well of the cell culture plate. Additional file 2: Table S1 shows required volumes of PDL stock (100 μg/mL) needed to coat different sizes of wells from various plates. PDL was on the plate overnight in the hood under UV light. On the day of the culture, the PDL was aspirated off the wells. The wells were rinsed with sterile water and the plates were dried under hood for at least 30 min. before plating the cells. It is important to rinse the wells with sterile water to completely remove residual PDL hydrobromide solution. Residual PDL can leave bromide salt on the plate, which is toxic to cells.

The dried PDL-coated plates can be stored at 4°C for 3–4 weeks. For plating cells on glass cover slips, autoclaved cover slips were placed using sterile forceps in wells with a diameter at least 1.25 times larger than the diameter of the cover slips. PDL coating was performed in the same way as mentioned above. Alternative to PDL, Poly-L-Ornithine, poly L-lysine or collagen can be used. However, poly L- Lysine is susceptible to degradation by the enzymatic actions of proteases released from the cells.

Vacuum adjustment in the sterile hood: It is important to reduce the suction pressure of the in-line vacuum or vacuum pump within the sterile hood. This vacuum line was used to aspirate media from the PET (at the time of culture preparation) or from the cell culture plates (on the day of the first media change) (see PROCEDURE). It is possible that the cell pellet/plated cells can be accidentally lost while aspirating the media if excessive vacuum force is used.

### Procedure

The overview of these steps is outlined in Figure 1.

#### Pre-culture preparation

The laminar cell culture hood surface was wiped with 70% ethanol and the germicidal UV light was turned on in the laminar flow hood. The autoclaved dissecting tools, sterile water, pipettes, pipettes tips, 100 mm cell culture plate were placed inside the cell culture hood and kept under the UV light for 30 min. A flat bucket with ice was made available just before the dissection. The culture media, sterile horse serum (Atlanta biologicals, cat no.S12150H) and trypsin-EDTA were pre-warmed at 37°C water bath. The tube containing the brain tissue in shipping media was removed from the shipping package and kept refrigerated.

It is important to maintain a record of the received brain tissues with detailed information (such as tissue code number, gender, gestational age, any birth defect, time of abortion).

#### Dissection/preparation of tissue

A 100 mm tissue culture plate containing 5 mL of shipping media was placed in the ice bucket. The brain tissue was transferred to the culture plate containing shipping media using the wide-bore siliconized Pasteur pipette. Any visible blood vessels and blood contaminated tissue were gently removed from the brain tissue mass. Effort should be made to have 7–15 gm. of cleaned tissue in order to yield sufficient number of cells.

The cleaned tissue was carefully chopped with the scalpel blade to create sections of approximately 0.5 mm thickness. It is important to note that the human samples are biohazards and personal protective equipment must be worn during dissection/preparation of the samples. Bleach solution was added to the plate containing removed tissue debris and tissue then discarded into the biohazard disposal container.

#### Enzymatic digestion of the brain tissue

The brain tissue mass was transferred into a 50 mL PET tube containing 10 mL of trypsin-EDTA and the tube was placed in the tube holder located in the water bath. The tube was oscillated the water bath at 150 RPM for 10–15 min.

The timing of enzymatic digestion is crucial for viability and yield of neuronal cells in the culture. We generally performed 15 min. digestion for brains that are obtained from the fetuses with gestational ages more than 105 days. Brains from lesser gestational age fetuses (typically less than 100 days) may require a 12 min. digestion. After the digestion, the tubes containing the brain tissue were wiped with 70% ethanol, brought back to the cell culture hood and 1 mL of sterile horse serum was immediately added to the trypsin-EDTA solution to stop the enzymatic reaction.

#### Isolation of cells from the tissue

The cell mass was transferred by the wide-bore glass Pasteur pipette from trypsin-EDTA to 15 mL PET tube, containing 5 mL of DM. It is convenient to equally distribute tissue into two separate 15 mL PET tubes, each containing 5 mL of DM. Dividing the tissue mass in this manner eliminates the need for balancing during centrifugation. Fire-polished siliconized Pasteur pipette was used to triturate the tissue with moderate force in the shipping media. Triturating 10–12 times is generally sufficient to obtain a fairly homogenous suspension of cells.

Vigorous or prolonged trituration can damage the cells resulting in lower/no cell yield. Some undigested mucinous brain tissue remnants are often present after trituration. Those to be removed from the tube and discarded into the appropriate biohazard waste container.

The tubes containing the brain homogenate were placed into the swinging tube holders of the centrifuge and centrifuged at 400 *g* at room temperature (22-27°C) for 5–8 minutes. Centrifugation will spin down all viable cells to the bottom of the tubes. After centrifugation, the tubes were gently removed from the centrifuge, wiped with 70% ethanol and placed in the sterile hood. The supernatant media was discarded using an untreated Pasteur pipette attached to the in-line vacuum.

Five (5) mL of fresh shipping media was poured into the tubes. The trituration step (as mentioned on above) was repeated once more, followed by further centrifugation at 400 *g* at room temperature for 5–8 minutes. After centrifugation, the supernatant media was gently aspirated out and add 3 mL of fresh shipping media was added into the tubes. The homogenate was mixed in the media by gently tapping the tubes or by slow pipetting with a P1000 micropipette. The cell suspensions from two different tubes were transferred into a single PET tube. The cell suspension (20 μL) was mixed with 180 μL of sterile DPBS (Cellgro, cat. no. 20-0131-CV; 1:100 dilutions). 10 μL of the diluted cell suspension was transferred into a sterile microfuge tube and 10 μL of trypan blue was and gently mixed and cell counting was carried out in a hemocytometer. Table 1 shows the average yield of cell number from Human fetal brain tissue samples that were obtained from aborted fetus of gestational age between 90–110 days. Aborted fetal brain tissues can be obtained from local abortion clinics or national distribution labs. In this study, we procured of cells obtained from brain tissue specimens. Cell counting can be performed in any automatic or semiautomatic cell counter following manufacturer’s instruction.

#### Cell plating

Cells (brain homogenate) were plated in PDL-coated plates (15000–25000 cells/cm [2]) using the defined culture medium (Neurobasal with supplements and antibiotics) with a P200 micropipette. Suggested volumes for plating are included in Additional file 1: Figure S1. Cell culture plates were swirled gently for uniform seeding of cells in the wells

Efforts should be made not to expose cells to ambient CO_2_ for more than 5 min. to minimize change in pH of the culture medium. It should be kept in mind that many cells in the fetal brain are NPCs, i.e. in presence of bFGF, these cells give rise to neurons and glia (Figures 2, 3 and 4). Plating too many cells can eventually result in excess cell density on the plate. Within 16–24 h of cell plating, the existing media was gently aspirated out using the in-line vacuum and replenish with freshly prepared defined culture medium. This step is important to remove unwanted cellular debris. Accumulation of cellular debris in the wells can retard cellular growth and also promote microbial contaminations. Reduced force of the in-line vacuum is necessary before aspirating the media.

#### Culture propagation

(i) 50% of the media was removed from the wells every 4th day and replaced by the same volume of media containing 2X growth factors until the termination of the culture. Representative morphology of cells at different time points is shown (Figure 2). Experiments on these cultures can be performed from 20–24 DIV onwards or earlier or later depending on the specific need of the experiments.

(ii) We have observed the presence of abundant neurons and neuronal protein markers even at DIV 40 of the culture. Absence of bFGF at early time points (up to DIV10-14) of the culture resulted in low cell yield. Withdrawal of bFGF at a later time point can make neurons degenerate gradually over time and can be used for specific drug studies [30].

#### Western immunoblotting

Western immunoblotting to detect proteins of interest was performed as previously described [31]. Briefly, culture cells were lysed by adding Mammalian Protein Extraction Reagent (M-PER; ThermoScientific; cat. no. 78501), supplemented with protease inhibitor cocktail directly to wells followed by shaking the plates on orbital shaker. Laemmli sample buffer was added to the cell lysates and boiled for 5 min. A list of antibodies used is provided in Table 2.

**Table 2:** **List of different primary and secondary antibodies used to characterize the culture**

**Primary antibody**	**Dilutions**	**Company**	**Cat.no.**	**Secondary antibody**	**Dilutions**	**Company**	**Cat.no.**
Panneuroral marker (ICC)	1:3000	Millipore	MAB2300	Biotinylated Donkey anti mouse	1:500	Jackson Immuno Research	715-065-150
Glial Fibrillary Acidic protein (GFAP) (WB)	1:20,000	Sigma	G9269	HRP-conj. Goat anti Rabbit	1:7,500	Thermo Scientific	31460
Glial Fibrillary Acidic protein (GFAP) (ICC)	1:10,000	Sigma	G9269	Cy3-conj. Donkey anti Rabbit	1:500	Jackson Immuno Research	711-165-152
Nestin (ICC)	1:1000	Sigma	N5413	Cy3-conj. Donkey anti Rabbit	1:500	Jackson Immuno Research	711-165-152
Neuron-specific Enolase (NSE) (WB)	1:3000	Abcam	ab16873	HRP-conj. Goat anti Rabbit	1:7,500	Thermo Scientific	31460
Synaptosomal-associated Protein of 25 kDa (SNAP25) (WB)	1:5000	Millipore	MAB331	HRP-conj. Goat anti Mouse	1:3000	Rockland	610-1319
Post Synaptic Density Protein of 95 kDa (PSD-95) (WB)	1:3000	NeuroMab	75-028	HRP-conj. Goat anti Mouse	1:3000	Rockland	610-1319
β actin (WB)	1:100,000	Sigma	A5441	HRP-conj. Goat anti Mouse	1:3000	Rockland	610-1319
5-Hydroxytryptamine (5-HT) (ICC)	1:1000	ImmunoStar	20080	Cy3-conj. Donkey anti Rabbit	1:500	Jackson Immuno Research	711-165-152
Glulamate Decarboxylase (GAD65/67) (WB)	1:1000	Millipore	AB1511	HRP-conj. Goat anti Rabbit	1:7,500	Thermo Scientific	31460
Tyrosine Hyroxylase (WB)	1:2000	Millipore	MAB318	HRP-conj. Goat anti Mouse	1:3000	Rockland	610-1319
Amyloid Precursor Protein (APP) (22C11 clone)	1:3000	Millipore	MAB348	HRP-conj. Goat anti Mouse	1:3000	Rockland	610-1319
α-tubulin	1:100,000	Sigma	T9026	HRP-conj. Goat anti Mouse	1:3000	Rockland	610-1319

#### Immunocytochemistry (ICC)

Cellular phenotypes were assessed by immunocytochemical (ICC), flow cytometry and confocal microscopy using neuronal antibody markers. For all these experiments, cells were fixed using paraformaldehyde (4%) and permeabilized by 0.13% triton X-100, followed by blocking with 10% horse serum per standard ICC protocols. Fixed cells were incubated overnight with neuron and glial-specific primary antibodies. Appropriate secondary antibodies and fluorophores were utilized to obtain fluorescence images as described before [4]. Figure 3 shows phenotypic character of the culture and also indicates that maturation of neurons (i.e. neurons and glial markers do not overlap) at DIV 24 onwards of the culture. Figure 6 shows presence of different types (for example, glutamatergic) of neurons at selected time points of the culture.

#### Transfection

Transfection of the cells was carried out using standard transfection reagent such as lipofectamine (for DNA constructs) and RNAiMAX (for small RNA). Transfection complexes were made using 50 μl/well Opti-MEM 1 (Gibco, Cat # 11058–021). Mock-transfected cells contained the Opti-Mem and 1. 75 μl/well RNAiMax, but no RNA. 20 nM APP siRNA was used to confirm transfectability of cells. The cells were transfected on DIV 19 at 37°C for 48 hours. After a PBS wash, 125 μL of M-per buffer (containing protease inhibitor) was used to lyse the cells (Figure 10).

#### Calcium imaging study

Calcium imaging was performed using Fura2-AM. Cells were incubated with Fura-2 AM for 1 h. After 3x PBS washes, the coverslips were placed in ‘standard extracellular solution buffer containing NaCl 130 mM, KCl 2 mM, MgCl2 1 mM, CaCl2 5 mM, glucose 5.5 mM, Hepes 10 mM, pH 7.2. 70 mM KCl was used to depolarize the cells. The baseline for all traces was transposed to allow comparison of peaks. T.I.L.L. Photonics (München, Germany) imaging system was used to obtain the data.

## Additional files

Additional file 1: Figure S1. The merged images from Figure 7 were auto-enhanced using ImageJ software. The top panel contains images from three different time points that are stained for a neuronal (Pan-N) and glial marker (GFAP). The bottom panel contains images from the same time points that are stained for neuronal (Pan-N) and neuronal progenitor (Nestin) marker. The auto-enhancement facilitates better visualization of the staining. However, it is not meant to be interpreted as indicative of staining across time points – as each image has its own auto-enhanced settings. Additional file 2: Table S1. Amount of poly-D-lysine required to coat different size of cell culture plates.

## Abbreviations

Aβ: Amyloid-β
AD: Alzheimer’s Disease
AM: Acetomethoxyl ester
APP: Amyloid precursor protein
BACE1: Beta-site APP-cleaving enzyme 1
BDNF: Brain-derived neurotrophic factor
bFGF: Basic fibroblast growth factor
DIV: Day in vitro
DM: Dissecting media
DPBS: Dulbecco’s phosphate-buffered saline
EDTA: Ethylenediaminetetraacetic acid
FURA 2-AM: Fura-2-acetomethoxyl ester
GDNF: Glial derived neurotrophic factor
GFAP: Glial fibrillary acidic protein
HFB: Human fetal brain
ICC: Immunocytochemistry
M-PER: Mammalian protein extraction reagent
NPC: Neuronal precursor cells
PC-12: Rat pheochromocytoma
PD: Parkinson’s disease
PDL: Poly-D-lysine
PSD-95: Post-synaptic density 95 kDa
NSE: Neuronal specific enolase
SK-N-SH: Human neuroblastoma cells
SNAP-25: Synaptosomal-associated protein 25 kDa
