# Supplementary figures and images for: Human primary mixed brain cultures: preparation, differentiation, characterization and application to neuroscience research
Source: Mol Brain. 2014 Sep 16;7:63. doi: 10.1186/s13041-014-0063-0 (PMC4181361; doi:10.1186/s13041-014-0063-0)

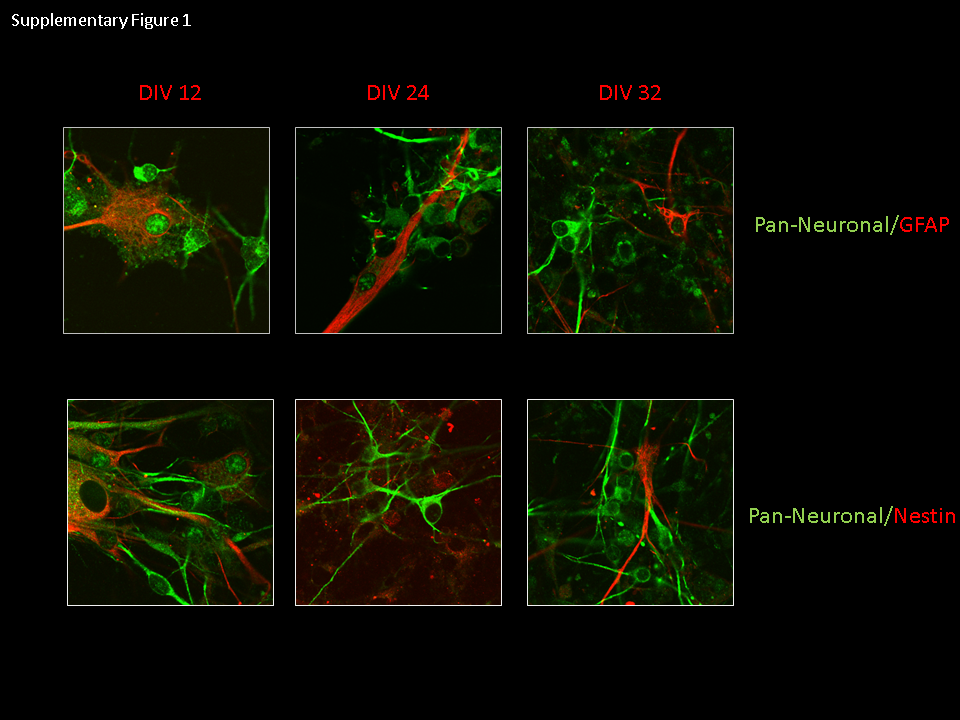

Supplement: Additional file 1: Figure S1. — The merged images from Figure 7 were auto-enhanced using ImageJ software. The top panel contains images from three different time points that are stained for a neuronal (Pan-N) and glial marker (GFAP). The bottom panel contains images from the same time points that are stained for neuronal (Pan-N) and neuronal progenitor (Nestin) marker. The auto-enhancement facilitates better visualization of the staining. However, it is not meant to be interpreted as indicative of staining across time points – as each image has its own auto-enhanced settings. [file 13041_2014_63_MOESM1_ESM.png]

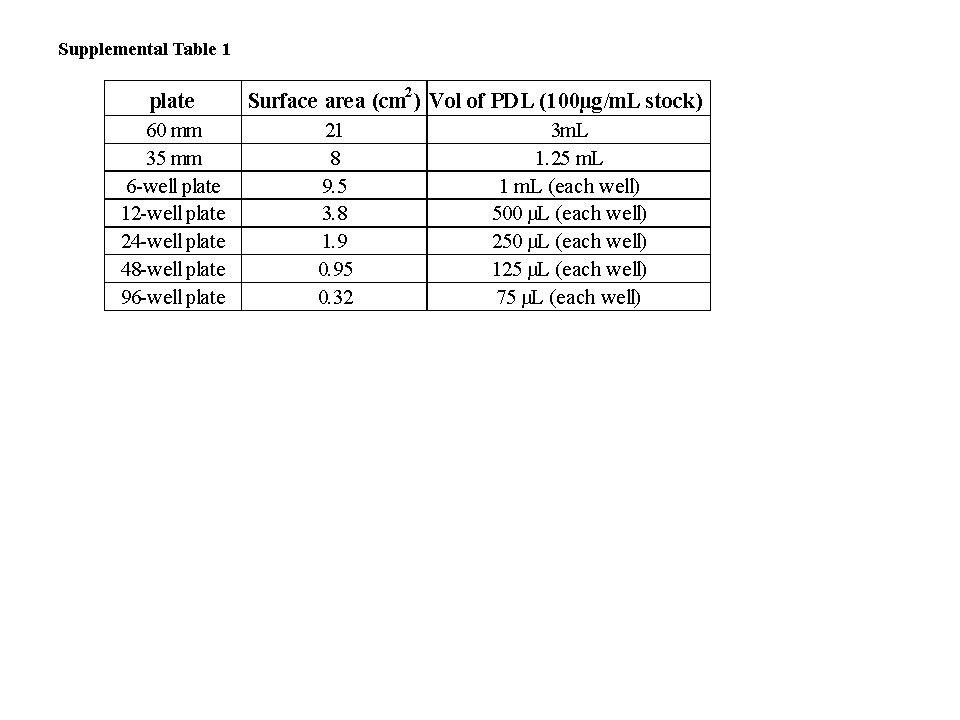

Supplement: Additional file 2: Table S1. — Amount of poly-D-lysine required to coat different size of cell culture plates. [file 13041_2014_63_MOESM2_ESM.png]
